# Construction of an EGFP-embedded porcine reproductive and respiratory syndrome virus infectious clone and antiviral drug screening

**DOI:** 10.3389/fcimb.2025.1653170

**Published:** 2025-08-26

**Authors:** Rongxiao Liu, Shi Fu, Yukun Chai, Nian Liu, Benjin Liu, Lingzhi Luo, Wei Yang, Xiumei Dong, Jin Cui

**Affiliations:** College of Veterinary Medicine, Northeast Agricultural University, Harbin, Heilongjiang, China

**Keywords:** PRRSV, BAC, EGFP, antiviral drug screening, reverse genetics system

## Abstract

Porcine reproductive and respiratory syndrome virus (PRRSV) is a significant pathogen posed a serious threat to the global swine industry. In this study, a BAC-based reverse genetics platform was established using a highly pathogenic PRRSV (HP-PRRSV) strain. Three recombinant reporter viruses were constructed by inserting the enhanced green fluorescent protein (EGFP) gene into three different intergenic regions of the complete PRRSV-L251 genome. Immunofluorescence assays combined with viral growth kinetics and reporter gene stability assessments indicated that rL251-ORF4-5a-EGFP maintained relatively stable expression during serial passage, and viral titers at 72 hours post-infection (hpi) were comparable to the parental virus. Subsequently, we identified four candidate compounds with potential anti-PRRSV activity using rPRRSV-L251-ORF4-5a-EGFP, indicating that this platform can be used as a visual assessment tool for antiviral drug screening. This study demonstrated that the ORF4-5a interval region is a feasible and promising site for exogenous gene insertion, and provided a robust technical platform for PRRSV vaccine development and pathogenesis studies.

## 1 Introduction

Porcine reproductive and respiratory syndrome (PRRS), caused by porcine reproductive and respiratory syndrome virus (PRRSV), is a viral disease characterized primarily by reproductive disorders in pregnant sows and respiratory symptoms in growing-finishing pigs. With high morbidity and mortality rates, PRRS has emerged as one of the most economically significant diseases affecting the global swine industry (Cai et al., 2023; Wensvoort et al., 1992). PRRSV is taxonomically classified under the family Arteriviridae, genus β-arterivirus, in the order Nidovirales (https://ictv.global). This non-segmented, single-stranded, positive-sense RNA virus possesses a genome approximately 15,000 nucleotides in length. The PRRSV genome comprises at least ten open reading frames (ORFs) arranged sequentially between the 5’UTR and 3’UTR as follows: ORF1a, ORF1b, ORF2a, ORF2b, ORF3 through ORF7, with certain adjacent ORFs exhibiting partial sequence overlap (Johnson et al., 2011; Lunney et al., 2016). ORF1a and ORF1b encode two polyproteins (pp1a and pp1ab) that undergo proteolytic processing by viral proteases to yield sixteen functionally diverse nonstructural proteins (Nsps). The remaining ORFs (ORF2a, ORF2b, ORF3-7, and ORF5a) encode eight structural proteins including GP2a, envelope protein (E), GP3, GP4, GP5, membrane protein (M), nucleocapsid protein (N), and GP5a (Dokland, 2010; Fang and Snijder, 2010; Music and Gagnon, 2010; Sun et al., 2023). This genomic organization and protein expression profile are fundamental to understanding PRRSV pathogenesis and developing effective control strategies.

Reverse genetics platform is a system that involves transfecting eukaryotic cells with constructs containing the full viral genome cDNA to restore infectious viral particles. When constructing infectious clones of PRRSV, most researchers use low-copy-number plasmids as vectors to ensure the genetic stability of infectious clones. Although these vectors can maintain genome stability, its limited capacity leads to genetic instability during exogenous gene replication, including genetic recombination, deletion, or mutation (Adler et al., 2003). The development of the bacterial artificial chromosome (BAC) system has solved this problem. BAC system has become the ideal vector for PRRSV infectious clone due to its high DNA capacity, excellent genetic stability, and strict copy number control (Li et al., 2022, 2015; Wang et al., 2013).

The genomic structural arrangements differ between the two PRRSV genotypes. In European strains, apart from the region between ORF1b and ORF2a, all adjacent open reading frames exhibit partial sequence overlap. In contrast, North American strains additionally possess a non-overlapping structure between ORF4 and ORF5 (Meulenberg et al., 1993; Nelsen et al., 1999). The presence of overlapping genes impedes N-terminal and C-terminal genetic analysis of structural proteins and complicates the insertion of foreign genes into the viral genome, thereby limiting the available insertion sites for exogenous gene manipulation. Previous study has identified several genomic loci suitable for foreign gene insertion in PRRSV, including the hypervariable region of Nsp2, the intergenic region between ORF1b-2a, ORF4-5a, and the junction between ORF7-3’UTR (Chaudhari and Vu, 2020). Utilizing these sites, researchers have successfully expressed various reporter genes (Han et al., 2017; Wang et al., 2014). However, stable expression of foreign genes relies critically on regulatory elements such as the transcription regulatory sequence (TRS). As a key cis-acting element in the PRRSV genome, TRS regulates discontinuous transcription. It features a highly conserved hexanucleotide core motif (5’-UUAACC-3’). Together with flanking sequences, this motif forms specific secondary structures that function as specialized regulatory cassettes, precisely guiding subgenomic mRNA synthesis (Pasternak et al., 2004, 2006).

Utilizing the highly pathogenic PRRSV-L251 strain as a model, we constructed a BAC-based infectious clone. Within this backbone, we inserted an EGFP reporter gene into three intergenic regions: ORF1b-ORF2a, ORF4-5a, and ORF7-3’UTR, and successfully rescued three corresponding reporter viruses. We demonstrated that the ORF4-5a IGR is a permissive site for stable foreign gene insertion. The rL251-ORF4-5a-EGFP reporter virus was obtained as a fluorescent-based high-throughput screening platform for antiviral discovery. Four small-molecule compounds with significant anti-PRRSV activity were identified using this platform.

## 2 Materials and methods

### 2.1 Cells, viruses and reagents

HEK-293T and Marc-145 cells were maintained in our laboratory. The HP-PRRSV-L251 strain (GenBank: PV631263) was isolated and propagated in our laboratory. DH10B competent cells were obtained from Shanghai Weidi Biotechnology Co., Ltd. The pBeloBAC11 vector was purchased from Hunan Fenghui Biotechnology Co., Ltd. Candidate antiviral compounds were procured from Meilun Biotechnology Co., Ltd. A polyclonal antibody against PRRSV-L251-N protein was generated in-house through murine immunization. The Multi-rAb™ CoraLite^®^ Plus 594-Goat Anti-Mouse Recombinant Secondary Antibody (H+L) was obtained from Proteintech Group, Inc. (Catalog No. RGAR004).

### 2.2 Plasmid construction

The pBeloBAC11 plasmid was linearized using HpaI and HindIII restriction enzymes. A CMV promoter fragment, amplified by PCR from the pCMV vector, was inserted into the linearized pBeloBAC11 via homologous recombination-based seamless cloning, generating the modified vector backbone (pBeloBAC-CMV) for PRRSV infectious clone construction. The full-length genomic cDNA of PRRSV-L251 was amplified as three overlapping fragments using gene-specific primers. The 3’UTR of PRRSV-L251, hepatitis delta virus ribozyme (Rz), and SV40 polyadenylation signal sequences were synthesized by Sangon Biotech. These components were sequentially assembled into the linearized pBeloBAC-CMV vector using homologous recombination-mediated seamless cloning to generate the full-length infectious clone.

Three intergenic regions, ORF1b-ORF2a, ORF4-ORF5a, and ORF7-3’UTR, were selected as candidate sites for EGFP reporter gene insertion. The EGFP gene was amplified with primers incorporating the TRS6 at its 5’- or 3’-terminus. Each EGFP-TRS6 cassette was inserted into the respective intergenic region of the linearized infectious clone plasmid via homologous recombination-based seamless cloning, generating the recombinant EGFP-tagged constructs.

### 2.3 Transfection and rescue of recombinant viruses

To rescue PRRSV/L251 and PRRSV-L251-EGFP viruses, the infectious clone plasmids were transfected into HEK-293T cells cultured in 24-well plates at 70-80% confluence using a DNA: PEI ratio of 1μg:3μL (optimized transfection ratio). Prior to transfection, DNA-PEI complexes were prepared as follows: 50 μL of Opti-MEM medium was aliquoted into a 1.5 mL EP tube, followed by addition of 800 ng infectious clone plasmid and corresponding 2.4 μL PEI transfection reagent (maintaining the 1μg:3μL ratio). After vortex mixing and brief centrifugation, the mixture was incubated at room temperature for 15 minutes The mixture was then added to cell culture medium with gentle shaking before continuing incubation. Approximately 48 hours post-transfection, cell culture supernatants were collected as P0 virus stock and stored at -80°C or directly used for subsequent experiments. Virus passage and amplification were performed by diluting P0 virus stock with DMEM medium containing 2% FBS, followed by inoculation (500 µL per well) onto confluent monolayers of Marc-145 cells. After 2 hours adsorption at 37°C, the viral inoculum was removed and replaced with fresh DMEM medium containing 2% FBS, followed by 72 hours incubation with observation for cytopathic effects (CPE). The harvested virus was serially passaged in Marc-145 cells using the same protocol, with each passage collected and stored at -80°C for subsequent experimental use.

### 2.4 Immunofluorescence assay

The infection status of parental PRRSV-L251 and recombinant reporter viruses in Marc-145 cells was assessed by IFA. Briefly, Marc-145 cells were seeded in 24-well plates and grown to confluent monolayers. The cells were then inoculated with viral supernatants. At 48 hpi, when CPE was observed, pre-cooled cell fixation solution was added to each well. The PRRSV-L251-N protein mouse polyclonal antibody was used as the primary antibody, while Multi-rAb™ CoraLite^®^ Plus 594-conjugated Goat Anti-Mouse Recombinant Secondary Antibody (H+L) served as the secondary antibody. Cell nuclei were counterstained with 4’,6-diamidino-2-phenylindole (DAPI). After staining, the results were observed and photographed under a fluorescence microscope.

### 2.5 Analysis of reporter virus stability and replication kinetics

The genetic stability of recombinant reporter viruses was assessed over serial passages. Viral supernatants from passages P0 to P10 were harvested and used to infect Marc-145 cells. At 72 hpi, EGFP expression and CPE were observed and recorded. Subsequently, cell supernatants were collected for RT-PCR analysis using specific primers to assess the stability of the EGFP gene across viral passages. Viral titers for each passage were determined using the Reed-Muench method.

To characterize the replication properties of reporter viruses during cultivation, *in vitro* replication kinetics of parental L251, rescued wild-type virus (rL251), and the reporter virus (rL251-ORF4-5a-EGFP) were compared in Marc-145 cells.

### 2.6 Antiviral drug screening

Cytotoxicity of six compounds (Curcumin, Pterostilbene, Brequinar, Isoliquiritigenin, β-Glycyrrhetinic acid, and Protocatechualdehyde) was assessed on Marc-145 cells using CCK-8 kit (Meilunbio, China). For antiviral screening, cells were infected with P3 of rL251-ORF4-5a-EGFP (MOI=0.1) and treated with different concentrations of compounds. After 72 h incubation, antiviral activity was evaluated by EGFP fluorescence quantification, TCID_50_ titration, and RT-qPCR.

### 2.7 Statistical analysis

Data were expressed as mean ± standard deviation (SD) from three independent experiments. Statistical comparison was analyzed by one-way analysis of variance (ANOVA). A value of P < 0.05 was considered statistically significant.

## 3 Result

### 3.1 The construction of infectious clones

**Figure 1:**
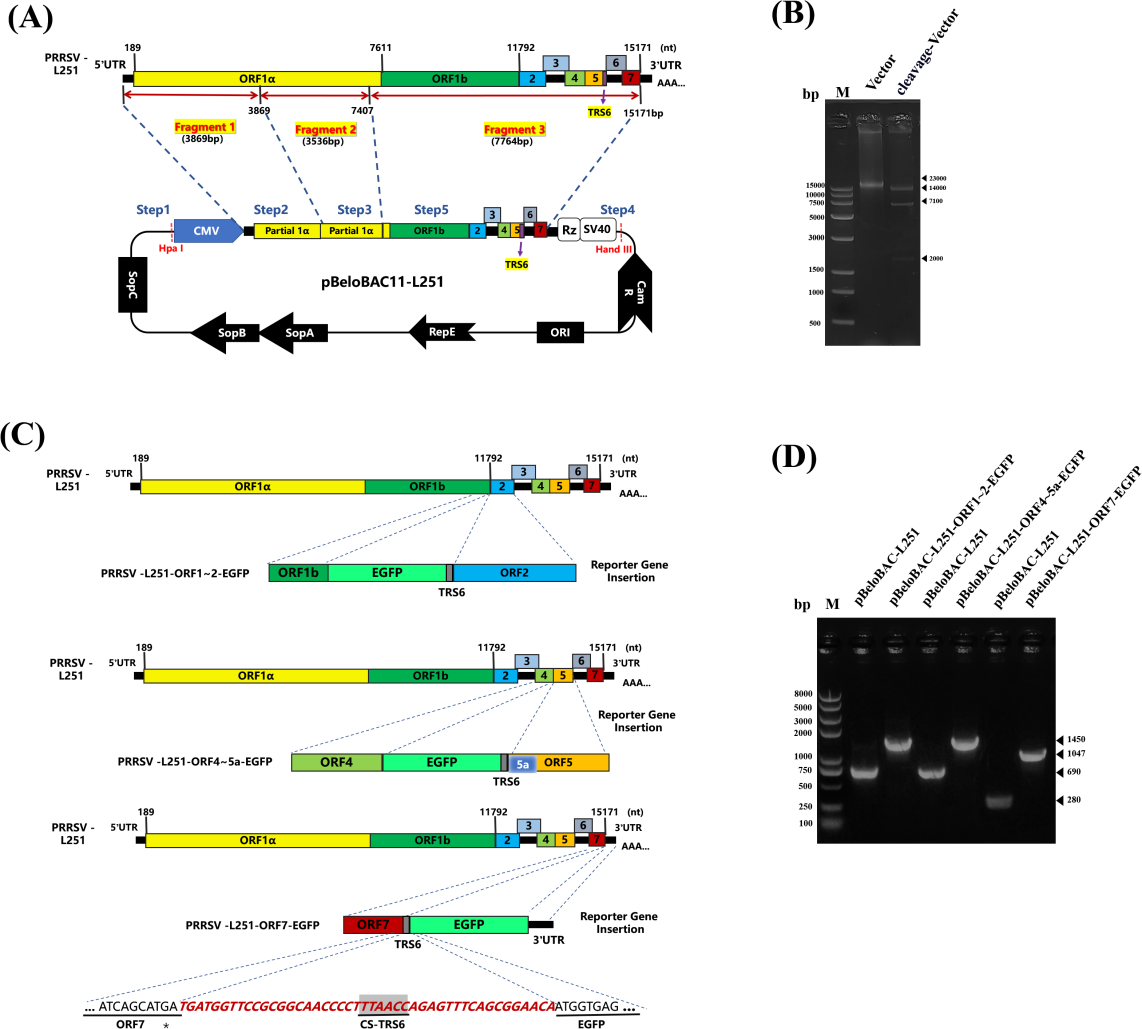
Construction and identification of PRRSV/L251 infectious clones **(A)**. Construction of PRRSV/L251 infectious clone. Three pairs of specific primers were designed based on the PRRSV-L251 strain genome sequence to amplify three cDNA fragments covering the complete viral genome by RT-PCR. Using homologous recombination technology, these fragments were sequentially inserted into the pBeloBAC recombinant vector. A cytomegalovirus (CMV) promoter sequence was inserted at the 5’ end, while the 3’ end contained synthesized components (3’ untranslated region, hepatitis delta virus ribozyme, and SV40 polyadenylation signal sequence) from Sangon Biotech to ensure efficient transcription and termination of the viral genome. **(B)** Restriction enzyme digestion analysis of pBeloBAC-L251. The recombinant plasmid was verified by double digestion with *NheI* and *SpeI* restriction endonucleases. Since the *SpeI* recognition site occurs twice in the plasmid, digestion yielded three DNA fragments of distinct sizes. **(C)** Construction of PRRSV-L251-EGFP infectious clones. Each EGFP construct contains TRS6 (transcription regulatory sequence, 5’-UUAACC-3’), where TRS6 inserted between ORF1b and ORF2a is located upstream of EGFP, and TRS6 inserted between ORF4 and ORF5a and between ORF7 and 3’UTR is located downstream of EGFP, to ensure coordinated transcription of the exogenous gene with the viral genome. The EGFP gene was inserted at three intergenic regions: between ORF1-ORF2, ORF4-5a, and ORF7-3’UTR, with a TRS6 sequence introduced either upstream or downstream of EGFP in each construct. **(D)** PCR verification of pBeloBAC-L251-EGFP. Using pBeloBAC-L251 as template, three pairs of specific primers were employed to amplify EGFP-containing regions for PCR-based validation of the recombinant constructs.

The infectious clone of PRRSV-L251 was constructed by using the BAC system. The sequences of CMV promoter, the 3’UTR of PRRSV, HDV ribozyme (Rz), and SV40 polyadenylation signal were sequentially inserted into pBeloBAC11, with the construction strategy shown in **Figure 1A**. The recombinant plasmid was verified by double enzyme reaction with *NheI* and *SpeI* restriction endonucleases (**Figure 1B**) and designated as pBeloBAC-L251.

To construct EGFP-chimeric infectious clones, EGFP gene fragments were amplified by PCR and inserted into the intergenic regions between ORF1-2, ORF4-5a, or ORF7-3’UTR in the pBeloBAC-L251 genome, respectively (**Figure 1C**). After PCR verification (**Figure 1D**), the recombinant plasmids were designated as pBeloBAC-L251-ORF1-2-TRS6-EGFP, pBeloBAC-L251-ORF4-5a-TRS6-EGFP, and pBeloBAC-L251-ORF7-3’UTR-TRS6-EGFP, respectively.

### 3.2 Rescue and identification of recombinant viruses

**Figure 2:**
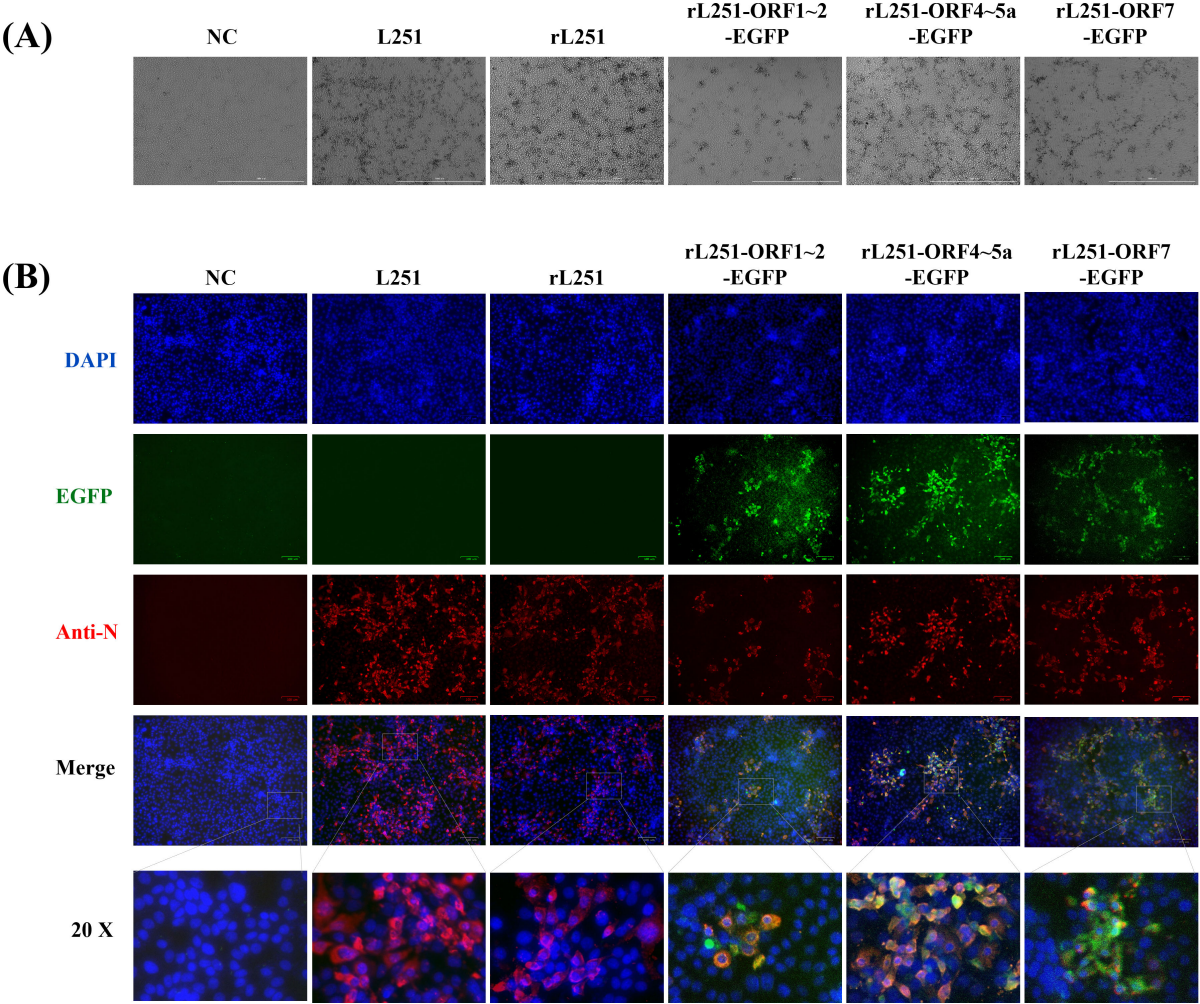
Viral rescue of rPRRSV/L251 and rPRRSV/L251-EGFP with IFA identification of N protein expression. **(A)**: CPE observation at 72 hpi for rL251 and rL251-EGFP on Marc-145 cells, with comparative analysis against the parental virus (L251)-induced CPE, while uninfected cells maintained normal morphology. **(B)**: Indirect immunofluorescence assay (IFA) of L251, rL251 and rL251-EGFP in Marc-145 cells at 72 hpi. Confluent MARC-145 cell monolayers were fixed and probed with N protein-specific polyclonal antibody as primary antibody, enabling simultaneous observation of both EGFP expression and specific fluorescence signals in L251, rL251- and rL251-EGFP-infected Marc-145 cells.

HEK-293T cells were transfected with infectious clone plasmids for 36 hours, and the supernatant was collected to infect Marc-145 cells. After 72 hours of culture, compared with the untreated group, the cells infected with recombinant virus exhibited the typical CPE of PRRSV infection (**Figure 2A**). Similarly, IFA results demonstrated efficient replication of recombinant viruses in Marc-145 cells, with a high degree of consistency between EGFP expression and viral N protein distribution, further confirming the association between EGFP expression and viral infection (**Figure 2B**).

### 3.3 Analysis of the growth characteristics of recombinant viruses

**Figure 3:**
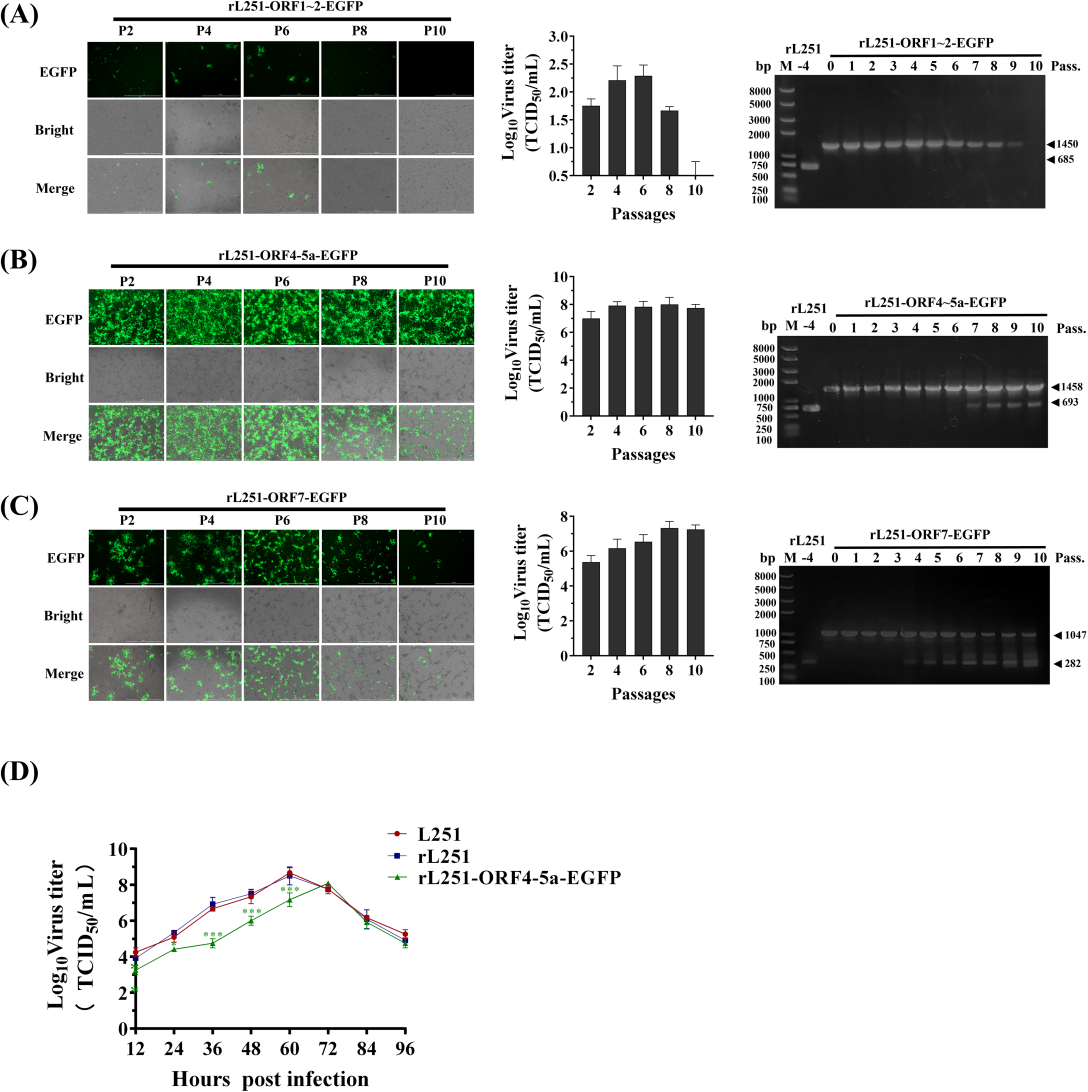
Stability and replication characteristics of reporter viruses **(A)**: Stability assessment of rL251-ORF1-2-EGFP. EGFP expression and CPE were observed by fluorescence microscopy in Marc-145 cells at 72 hpi across multiple passages. Viral supernatants were collected for titer determination (TCID_50_), while cell pellets were subjected to RT-PCR analysis. **(B)**: Stability assessment of rL251-ORF4-5a-EGFP. EGFP expression and CPE were monitored by fluorescence microscopy in Marc-145 cells at 72 hpi through serial passages. Viral titers were determined from collected supernatants, with parallel RT-PCR analysis of cell pellets. **(C)**: Stability assessment of rL251-ORF7-EGFP. Fluorescence microscopy tracked EGFP expression and CPE development in Marc-145 cells at 72 hpi during sequential passaging. Supernatants were assayed for viral titers, while cell precipitates underwent RT-PCR detection. **(D)**: Multi-step growth curve analysis of L251, rL251, and rL251-EGFP. P3 virus was used to infect cells, with viral titers (log10TCID_50_/mL) determined at 0, 12, 24, 36, 48, 60, 72, 84, and 96 hpi to generate replication kinetics.

Verification of the stability of the EGFP gene in the genomes of the three reported viruses showed that rL251-ORF1-2-EGFP exhibited the highest genetic stability, with PCR products of each generation of the virus maintaining the expected size (1450 bp) and no abnormal bands appearing. However, this viral strain exhibited significant replication defects during serial passage. The results indicated that insertion of an exogenous gene into the ORF1–2 region can maintain genetic stability but may severely impair viral replication function (**Figure 3A**). rL251-ORF7-EGFP began to exhibit genetic instability after P4, maintaining strong EGFP expression during early passages (P0–P3), but with increasing passage, EGFP gene loss became increasingly evident. Concurrently, viral titers showed an upward trend, suggesting that EGFP gene loss may have restored partial replication capacity (**Figure 3B**).

rL251-ORF45a-EGFP exhibited unique biological characteristics. It demonstrated high genetic stability from P0 to P6, and viral titer assays indicated that its replication capacity was significantly superior to that of the other two reporter viruses (**Figure 3C**). Next, viral titers of L251, rL251, and rL251-ORF45a-EGFP (P3) were measured at different time points, and growth kinetic curves were generated. The results showed that the replication profiles of L251 and rL251 were nearly identical (**Figure 3D**). Based on these findings, the P3 of rL251-ORF4-5a-EGFP was established as the standard working viral strain for subsequent experiments.

### 3.4 Antiviral drug screening

**Figure 4:**
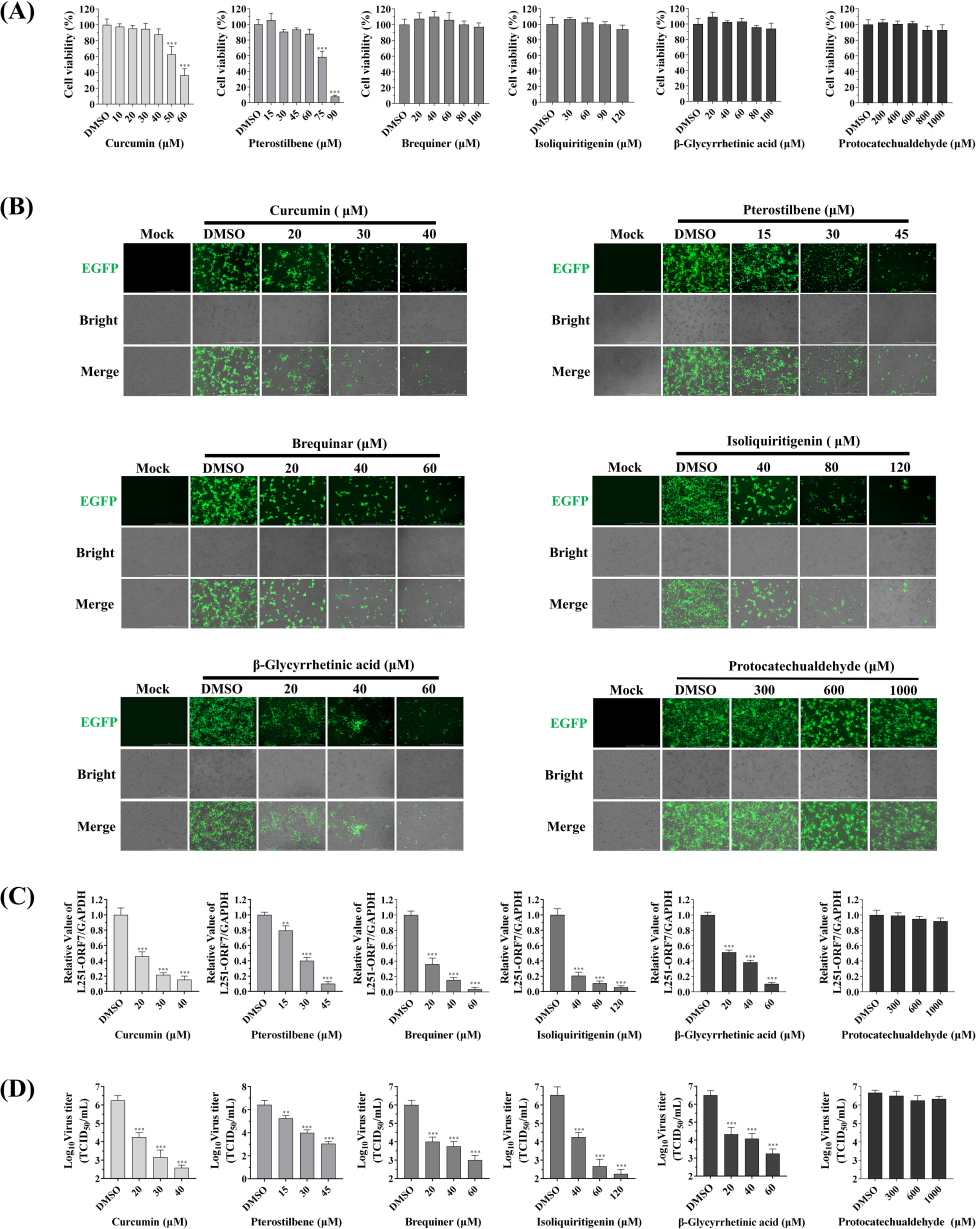
Screening of anti-PRRSV compounds. **(A)**: Determination of MNTC for six small-molecule compounds in Marc-145 cells. Cytotoxicity was assessed using Cell Counting Kit-8 assay. Compounds were serially diluted in DMEM with 2% FBS across concentration gradients (100 μL/well, triplicate wells per concentration). Control groups included: untreated cell control and blank control. Absorbance was measured at 450 nm using a microplate reader. **(B)**: Evaluation of EGFP fluorescence signal inhibition by six compounds against PRRSV *in vitro*. Compounds were diluted in DMEM with 2% FBS to concentrations at/below MNTC (100 μL/well, triplicate wells per concentration). Control groups included: virus-infected cells with 1% DMSO in 2% FBS DMEM (DMSO control), and uninfected cells with 1% DMSO in 2% FBS DMEM (blank control). After 48h incubation, EGFP signals and CPE were documented. **(C)**: RT-qPCR analysis of antiviral effects. Total RNA was extracted from compound-treated and virus control groups after 48h treatment. PRRSV-L251-ORF7-specific primers were used for real-time PCR (triplicate technical replicates per sample). **(D)**: Viral titer determination by endpoint dilution assay. Supernatants from treated and control groups were serially diluted (10-fold gradients) and inoculated onto Marc-145 cells. After 72h incubation, CPE was recorded, and TCID_50_/mL was calculated using Reed-Muench method. Statistical comparison was analyzed by ANOVA (n=3, P>0.05, not significant; *P<0.05; ***P<0.001).

In the antiviral activity evaluation experiments, the maximum non-toxic concentrations of six compounds were first determined (**Figure 4A**). The treatment groups with Curcumin, Pterostilbene, Brequinar, Isoliquiritigenin and β-Glycyrrhetinic acid all exhibited significant dose-dependent anti-PRRSV effects. Fluorescence microscopy observation revealed that when drug concentrations reached 40μM or below, the EGFP fluorescence signal intensity of rL251-ORF4-5a-EGFP reporter virus showed obvious reduction (**Figure 4B**). Quantitative analysis revealed that RT-qPCR-detected viral gene decreased by ~20% (Pterostilbene) to 80% (Isoliquiritigenin) (**Figure 4C**). Viral titer determination results also showed significant reduction compared to the DMSO control group; when drug concentrations were increased to maximum levels, the antiviral effects became more pronounced (**Figure 4D**). The Protocatechualdehyde treatment group did not exhibit dose-dependent antiviral effects. Systematic testing revealed no significant differences between the 1000μM drug test group and the DMSO control group.

## 4 Discussion

Previous studies have demonstrated significant sequence variability in the Nsp2 protein, with amino acid homology ranging only from 66 to 70% among strains within the same PRRSV subtype and approximately 30% between different subtypes (Han et al., 2006). Several studies have revealed that the hypervariable region (HVR) of Nsp2 can naturally accommodate nucleotide insertions and deletions (Gao et al., 2004; Han et al., 2006; Ni et al., 2011; Shen et al., 2000). Deletions in the N-terminal 13-35aa and the 324-813aa regions of HVR appear to be tolerated. Within the HVR, the maximum permissible deletion around positions 324-726aa is approximately 400aa, although deletions up to 200aa are more compatible with maintaining viral infectivity (Han and Yoo, 2014; Zhou et al., 2024). These characteristics suggest that Nsp2 represents a potential site for insertion of foreign marker proteins (Kim et al., 2007). Xu YZ et al. successfully inserted a 49aa sequence encoding part of the Newcastle disease virus nucleoprotein (NDV NP) gene into the Nsp2 region of the Hun4-F112 strain, which remained stably expressed even after 20 consecutive passages. However, subsequent attempts to insert various other genes at different positions within the Nsp2 region mostly failed to rescue viable viruses, indicating that while the NSP2 region possesses some capacity for foreign gene insertion, its tolerance is highly dependent on the specific sequence inserted (Xu et al., 2012). Similarly, GFP expression is unstable during serial passaging after inserting GFP between amino acids 324 and 434 of Nsp2 in the PRRSV-VR-2332 strain (Han et al., 2007). Pei Y et al. successfully inserted both the EGFP gene and porcine circovirus type 2 capsid protein gene between ORF1b and ORF2a of the P129 strain, with TRS6 incorporated downstream of EGFP to regulate exogenous gene expression while TRS2 controlled downstream ORF2 expression. This engineered recombinant virus maintained stable EGFP expression through 37 consecutive passages (Pei et al., 2009).

Building upon these findings, we established a Bacterial Artificial Chromosome (BAC)-based reverse genetics platform for the highly pathogenic PRRSV L251 strain and constructed EGFP-tagged reporter viruses targeting three distinct genomic regions. Our data revealed that EGFP insertion at the ORF1b-ORF2a junction severely impaired viral replication. During serial passaging (P1-P10), the rL251-ORF1-2-EGFP reporter virus induced minimal CPE in Marc-145 cells and exhibited markedly weaker EGFP fluorescence compared to other constructs. This defect likely arises from EGFP-induced genomic rearrangements disrupting normal viral RNA transcription/translation or substantially interfering with viral particle assembly during replication, though further experimental validation is required. For the rL251-ORF7-EGFP reporter virus constructed at the ORF7-3’UTR junction, while maintaining robust EGFP expression during early passages (P0-P3), significant gene loss became evident from P4 onward. Interestingly, viral titers increased with successive passages, suggesting an inverse correlation between EGFP retention and replication competence.

The reporter virus rL251-ORF4-5a-EGFP, demonstrated relative stable EGFP expression. Compared to the unstable ORF4-5a EGFP virus which utilized native TRS5 upstream of ORF5 and lost the EGFP insert by passage 8 (P8) (Wang et al., 2020), our approach strategically employed TRS6. TRS6 features a simpler RNA secondary structure and shorter flanking sequences. This optimization enabled sustained EGFP expression through 10 consecutive passages (P10). Our system could be further optimized regarding insertion length. Longer exogenous genes, such as the 720 bp EGFP insert, negatively correlate with PRRSV stability during passage. Consequently, employing smaller tags may enhance stability by reducing genomic burden, a direction for future optimization studies.

This study delivers three principal advances: (1) Development of the first BAC-based reverse genetics system for HP-PRRSV strain L251, utilizing the high capacity and low copy number properties of bacterial artificial chromosomes to resolve genomic instability typical of plasmid-based approaches; (2) Optimization of the ORF4-5a intergenic region as a stable insertion site via the TRS6 regulatory element, achieving sustained EGFP expression through P10 passage (>50% retention after P7 deletion), contrasting with complete EGFP loss by P8 using TRS5 in the identical region; and (3) Functional application of the rL251-ORF4-5a-EGFP reporter virus for real-time infection visualization and high-throughput screening, identifying four novel anti-PRRSV compounds. Collectively, these innovations establish a robust platform for antiviral development and a foundational framework for PRRSV vector engineering.

In conclusion, this study successfully established a reverse genetics system for PRRSV based on the BAC vector, and rescued an EGFP reporter virus exhibiting favorable genetic stability. The developed platform offers a visual evaluation tool for subsequent investigations into PRRSV pathogenesis, neutralization assays, and antiviral compound screening.

## Data Availability

The original contributions presented in the study are included in the article/Supplementary Material. Further inquiries can be directed to the corresponding authors.
